# Micro-Doppler Effect Removal in ISAR Imaging by Promoting Joint Sparsity in Time-Frequency Domain

**DOI:** 10.3390/s18040951

**Published:** 2018-03-23

**Authors:** Lin Sun, Weidong Chen

**Affiliations:** Key Laboratory of Electromagnetic Space Information of the Chinese Academy of Sciences, University of Science and Technology of China, Hefei 230027, China; sunlin@mail.ustc.edu.cn

**Keywords:** inverse synthetic aperture radar imaging, micro-Doppler effect, joint sparsity, short time Fourier transform, time-frequency domain

## Abstract

For micromotion scatterers with small rotating radii, the micro-Doppler (m-D) effect interferes with cross-range compression in inverse synthetic aperture radar (ISAR) imaging and leads to a blurred main body image. In this paper, a novel method is proposed to remove the m-D effect by promoting the joint sparsity in the time-frequency domain. Firstly, to obtain the time-frequency representations of the limited measurements, the short-time Fourier transform (STFT) was modelled by an underdetermined equation. Then, a new objective function was used to measure the joint sparsity of the STFT entries so that the joint sparse recovery problem could be formulated as a constrained minimization problem. Similar to the smoothed $l_{0}$ (SL0) algorithm, a steepest descend approach was used to minimize the new objective function, where the projection step was tailored to make it suitable for m-D effect removal. Finally, we utilized the recovered STFT entries to obtain the main body echoes, based on which cross-range compression could be realized without m-D interference. After all contaminated range cells were processed by the proposed method, a clear main body image could be achieved. Experiments using both the point-scattering model and electromagnetic (EM) computation validated the performance of the proposed method.

## 1. Introduction

In inverse synthetic aperture radar (ISAR) imaging, mechanical rotations or vibrations of structures on a target may introduce additional frequency modulations on the returned signal, known as the micro-Doppler (m-D) effect [1,2,3]. The main body image is usually blurred because of the interference of the rotating or vibrating scatterers, which are also called micromotion scatterers [3]. Therefore, the m-D effect should be removed in order to obtain a clear image of the main body.

Micromotion targets with rotating structures can often be found in real world scenarios, e.g., a ship with scanning antennas or an aircraft in flight equipped with turbofans. A micromotion scatterer with a large rotating radius will generate a m-D signal which can be depicted in the form of sinusoidal modulations in the spectrogram after range compression [4,5]. In contrast, the main body signal in the spectrogram has the shape of straight lines [5]. Based on the different shapes in the spectrogram, many methods [4,5,6,7] have been proposed to eliminate the m-D signal. Li and Ling [4] put forward an adaptive chirplet decomposition algorithm in which the returned signal is decomposed into a family of chirplet functions. The main body signal and the micromotion signal subsequently are separated according to their distinct chirp rates. However, this algorithm has high computation cost because of the large chirplet dictionary. To remove the sinusoidal m-D interference, the method in Q. Zhang et al. [6] extracts the straight lines in the spectrogram using the Hough transform. Similarly, the methods in H.C. Liu et al. and L. Sun et al. [5,7] only recover the straight lines via the sparse representation (SR)-based algorithms where the sinusoids are eliminated. Nevertheless, the aforementioned methods [5,6,7] cannot remove the m-D effect generated by a micromotion scatterer with a small rotating radius, since the m-D signal has the same straight line shape as the main body signal in the spectrogram when the rotating radius is smaller than half of the range resolution.

Recently, some methods based on the time-frequency analysis have been developed to address the problem of the removal of the m-D effect under a small rotating radius. L. Stanković et al.’s method [8], based on L-statistics, performs the short-time Fourier transform (STFT) to the echoes in the contaminated range cell. It is assumed in L. Stanković et al. [8] that the m-D interference occupies a small portion of time instants at each frequency bin. Using the L-statistics, a fixed fraction of the most significant STFT entries were eliminated to achieve m-D effect removal. Subsequently, the remaining entries were utilized to recover the main body signal. However, a large amount of STFT entries corresponding to the main body might be removed together with the m-D interference, which leads to a high sidelobe level in the imaging result [9]. A method based on histogram analysis is proposed in R. Zhang et al. [9], where the STFT entries with a high frequency of occurrence were regarded as the main body components and were preserved to reconstruct the ISAR image. On the other hand, the STFT entries with a low frequency of occurrence were considered to correspond to the m-D interference and suppressed. When the m-D effect is severe, the method in R. Zhang et al. [9] cannot remove the m-D signal in the time-frequency domain completely because the m-D components might be mistaken for the main body signal. Consequently, there are some spurious points in the imaging result due to the residual m-D signal. In real world situations, the data samples of the echoes might be randomly missing at given time instants [10] where strong electromagnetic interference or sensor failure prevents effective observation. As a result, the effective pulses are limited. The L-statistics-based method is combined with the short-time compressed sensing (STCS) [11] approach in Q.K. Hou et al. [12] to reduce the m-D effect with limited pulses. The STCS was employed to recover the STFT entries where the number of the frequency bins was equal to the number of full pulses. Nevertheless, the window width in the STCS could have been much shorter than the signal duration to obtain the local frequency characteristics. Thus, the measurements in the window were much fewer than the frequency bins in the time-frequency domain. The frequency resolution of the recovered STFT entries was reduced due to the extremely limited measurements. As a result, the L-statistics-based method in Q.K. Hou et al. [12] cannot obtain the accurate support of the main body signal in the frequency dimension and some spurious points exist near the main body scatterers in the imaging result.

The main body scatterer has a constant Doppler frequency [13]. Thus, the support of the main body signal is a slow time invariant in the time-frequency domain which indicates the joint sparsity [14,15] of the main body signal. In contrast, the Doppler frequency of the micromotion scatterer varies with the slow time [13]. Based on the distinct patterns in the time-frequency domain, this paper proposes a joint sparsity-based ISAR imaging method to remove the m-D effect generated by the micromotion scatterers with small rotating radii. Firstly, the echoes in the contaminated range cell were modelled by an underdetermined equation where the relation of the echoes and the corresponding STFT entries is built on the STFT matrix. Generally, the $l_{2}/l_{0}$-norm [16] of the STFT entry vector could be used to measure the joint sparsity in the time-frequency domain because it is equal to the number of frequency bins having nonzero STFT entries. Nonetheless, minimizing the $l_{2}/l_{0}$-norm leads to a combinatorial optimization problem [17] that is difficult to solve. In M. Bevacqua et al. [18], the $l_{1}$-norm of an auxiliary variable, defined as the common upper bound to the amplitudes of the electric currents, is utilized to replace the $l_{2}/l_{0}$-norm so that the new problem is tractable. However, the difference between the $l_{1}$-norm of the auxiliary variable and the original $l_{2}/l_{0}$-norm is significant [19] because larger coefficients are penalized more heavily than smaller coefficients in the $l_{1}$-norm. A reweighted $l_{1}$-norm method is proposed in Y. Liu et al. [19] to overcome the drawback of the $l_{1}$-norm, but this method is lack of convergence guarantee [20]. Recently, the $l_{2}/l_{1}$-norm was utilized to promote the joint sparsity of highly conductive scatterer in microwave imaging [21]. Nonetheless, the $l_{2}/l_{1}$-norm is a loose approximation to the original $l_{2}/l_{0}$-norm and often leads to suboptimal solutions [22]. Different from the methods in M. Bevacqua et al., Y. Liu et al., and S. Sun et al. [18,19,21], we propose a new objective function in this paper which can achieve tight approximation to the original $l_{2}/l_{0}$-norm with proper parameters. The new objective function was minimized, similar to the smoothed $l_{0}$ (SL0) algorithm approach [23] which consisted of two steps, i.e., the steepest descend step and the projection step. Since the m-D signal was suppressed in the steepest descend step, in this paper, the projection step of SL0 was tailored to make it suitable for m-D effect removal. By minimizing the new objective function, the joint sparsity in the time-frequency domain was promoted and the m-D interference could be effectively removed. Subsequently, we utilized the recovered STFT entries to obtain the main body echoes free of m-D effect. Finally, the cross-range compression [24] of the main body echoes was realized through the sparse Bayesian learning (SBL) algorithm [25]. When the m-D effect in all of the contaminated range cells was removed by the proposed method, a clear main body image could be achieved.

The rest of this paper is organized as follows. Section 2 presents the signal model of ISAR imaging for micromotion targets with rotating parts. Section 3 demonstrates the joint sparsity of the main body signal in the time-frequency domain. The proposed ISAR imaging method is elaborated in Section 4, where we introduce the joint sparse recovery problem based on the $l_{2}/l_{0}$-norm and briefly review the SL0 algorithm. In Section 5, we discuss experiments based on the point-scattering model and electromagnetic (EM) computation which were conducted to validate the effectiveness of the proposed method. Finally, conclusions are drawn in Section 6.

We introduce the following notations in this paper. Bold uppercase letters and bold lowercase letters are reserved for matrices and vectors, respectively. $A\in {\mathbb{C}}^{M\times N}$ denotes a matrix of size M×N with complex elements. ${\left\|A\right\|}_{F}$ denotes the Frobenius norm of A. For a vector x, its ith element is denoted by $x_{i}$. ${\left\|x\right\|}_{0}$, ${\left\|x\right\|}_{1}$, and ${\left\|x\right\|}_{2}$ represent the $l_{0}$-norm, $l_{1}$-norm and $l_{2}$-norm of x, respectively. ${\left(\cdot \right)}^{T}$ and ${\left(\cdot \right)}^{H}$ stand for the transpose and the conjugate transpose of a matrix or a vector.

## 2. ISAR Imaging Model

**Figure 1:**
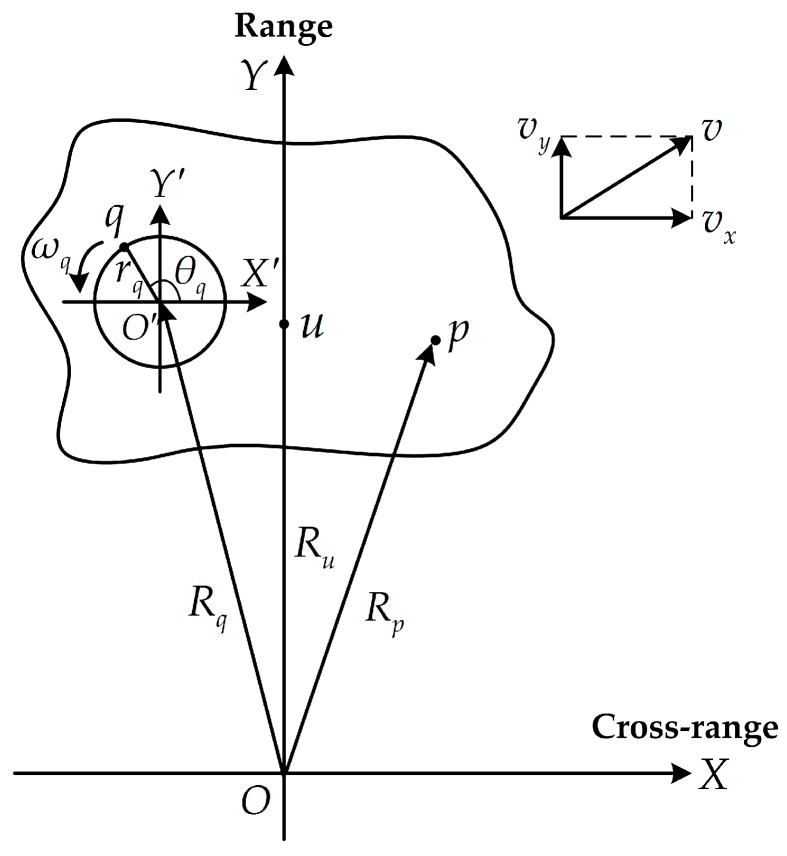
Inverse synthetic aperture radar (ISAR) imaging geometry with rotating scatterers.

To simplify the analysis, we consider the point-scattering model [4] to illustrate the m-D effect. As shown in Figure 1, the radar is located at the origin O of the coordinate system XOY. The target center $u\left(x_{u},\;y_{u}\right)$ is located at the Y-axis which indicates the line of sight (LOS). Without loss of generality, we assume that the target moves within the 2-dimensional (2-D) imaging plane XOY with velocity v and the motion compensation [26] has been accomplished. The projection of v along the direction of the X-axis is denoted by $v_{x}$ which generates the aspect angle variation utilized in ISAR imaging. $p\left(x_{p},\;y_{p}\right)$ and $q\left(x_{q},\;y_{q}\right)$ denote the main body scatterer and the micromotion scatterer, respectively. $O^{\prime }\left(x_{o^{\prime }},\;y_{o^{\prime }}\right)$ is the rotating center of the micromotion part. q rotates around $O^{\prime }$ with radius $r_{q}$, angle frequency $\omega_{q}$, and initial rotation angle $\theta_{q}$. At the initial processing time, the distances from p, q, and u to the radar are $R_{p}$, $R_{q}$, and $R_{u}$, respectively.

The transmitted chirp signal is

(1) $$s\left(\hat{t},t_{m}\right)=\mathrm{rect}\left(\frac{\hat{t}}{T}\right)\exp\left(j2\pi \left(f_{c}\left(\hat{t}+t_{m}\right)+\frac{1}{2}\gamma {\hat{t}}^{2}\right)\right)$$

where $\hat{t}$ and $t_{m}$ denote the fast time and the slow time, respectively. T is the pulse duration, $f_{c}$ is the carrier frequency, and γ is the chirp rate. rect(⋅) represents the rectangular function, which is defined as

(2) $$\mathrm{rect}\left(\frac{\hat{t}}{T}\right)=\left\{ \begin{array}{ll} 1, & \left|\hat{t}\right|\leq T/2\\ 0, & \left|\hat{t}\right|>T/2. \end{array}\right.$$

To reduce the received effective bandwidth, the dechirp method is applied. Taking the target center as the reference point in the dechirping process, the reference signal is given by

(3) $$s_{ref}\left(\hat{t},t_{m}\right)=\mathrm{rect}\left(\frac{\hat{t}-2\frac{R_{u}\left(t_{m}\right)}{c}}{T_{ref}}\right)\exp\left(j2\pi \left(f_{c}\left(\hat{t}-2\frac{R_{u}\left(t_{m}\right)}{c}+t_{m}\right)+\frac{1}{2}\gamma {\left(\hat{t}-2\frac{R_{u}\left(t_{m}\right)}{c}\right)}^{2}\right)\right)$$

where $T_{ref}$ is the duration of the reference signal with $T_{ref}>T$, and c denotes the speed of light. After dechirping and removing the residual video phase, the echo can be expressed as

(4) $$\begin{array}{ll} s_{r}\left(\hat{t},t_{m}\right) & =\sum_{p=1}^{P}\sigma_{p}\mathrm{rect}\left(\frac{\hat{t}-2\frac{R_{p}\left(t_{m}\right)}{c}}{T}\right)\exp\left(-j\frac{4\pi }{c}\left(f_{c}+\gamma \left(\hat{t}-2\frac{R_{u}\left(t_{m}\right)}{c}\right)\right)\Delta R_{p}\left(t_{m}\right)\right)\\ & +\sum_{q=1}^{Q}\sigma_{q}\mathrm{rect}\left(\frac{\hat{t}-2\frac{R_{q}\left(t_{m}\right)}{c}}{T}\right)\exp\left(-j\frac{4\pi }{c}\left(f_{c}+\gamma \left(\hat{t}-2\frac{R_{u}\left(t_{m}\right)}{c}\right)\right)\Delta R_{q}\left(t_{m}\right)\right) \end{array}$$

where $\sigma_{p}$ and $\sigma_{q}$ denote the backscattering coefficients of the pth main body scatterer and the qth micromotion scatterer, respectively. After the Fourier transform to Equation (4) along the $\hat{t}$ dimension, the range compression is achieved and the spectrogram can be written as

(5) $$\begin{array}{ll} s_{r}\left(\hat{f},t_{m}\right) & =\sum_{p=1}^{P}A_{p}\sin c\left(T\left(\hat{f}+2\frac{\gamma }{c}\Delta R_{p}\left(t_{m}\right)\right)\right)\exp\left(-j\frac{4\pi }{\lambda }\Delta R_{p}\left(t_{m}\right)\right)\\ & +\sum_{q=1}^{Q}A_{q}\sin c\left(T\left(\hat{f}+2\frac{\gamma }{c}\Delta R_{q}\left(t_{m}\right)\right)\right)\exp\left(-j\frac{4\pi }{\lambda }\Delta R_{q}\left(t_{m}\right)\right) \end{array}$$

where $\hat{f}$ is the fast frequency and λ is the wavelength. $A_{p}=\sigma_{p}T$ and $A_{q}=\sigma_{q}T$ denote the complex coefficients of scatterers p and q, respectively.

It is assumed that the target has a constant velocity $v_{x}$ in a short coherent processing interval (CPI). Thus, the aspect angle variation of the target is calculated to be $v_{x}t_{m}/R_{u}$. Because the target is far from the radar, the instantaneous distances from p and q to the reference point can be approximately expressed as

(6) $$\Delta R_{p}\left(t_{m}\right)=x_{p}\frac{v_{x}t_{m}}{R_{u}}+y_{p}-y_{u}$$

(7) $$\Delta R_{q}\left(t_{m}\right)=x_{o^{\prime }}\frac{v_{x}t_{m}}{R_{u}}+y_{o^{\prime }}-y_{u}+r_{q}\sin\left(\omega_{q}t_{m}+\theta_{q}\right).$$

Substituting Equations (6) and (7) into Equation (5) yields

(8) $$\begin{array}{l} s_{r}\left(\hat{f},\;t_{m}\right)\approx \sum_{p=1}^{P}A_{p}\sin c\left(T\left(\hat{f}+\frac{2\gamma }{c}\left(y_{p}-y_{u}\right)\right)\right)\exp\left(-j\frac{4\pi }{\lambda }\left(x_{p}\frac{v_{x}t_{m}}{R_{u}}+y_{p}-y_{u}\right)\right)\\ \;+\sum_{q=1}^{Q}A_{q}\sin c\left(T\left(\hat{f}+\frac{2\gamma }{c}\left(y_{o^{\prime }}-y_{u}+r_{q}\sin\left(\omega_{q}t_{m}+\theta_{q}\right)\right)\right)\right)\exp\left(-j\frac{4\pi }{\lambda }\left(x_{o^{\prime }}\frac{v_{x}t_{m}}{R_{u}}+y_{o^{\prime }}-y_{u}+r_{q}\sin\left(\omega_{q}t_{m}+\theta_{q}\right)\right)\right). \end{array}$$

It can be seen from Equation (8) that the peaks of the spectrogram are located at

(9) $${\hat{f}}_{p}=-\frac{2\gamma }{c}\left(y_{p}-y_{u}\right)$$

(10) $${\hat{f}}_{q}=-\frac{2\gamma }{c}\left(y_{o^{\prime }}-y_{u}+r_{q}\sin\left(\omega_{q}t_{m}+\theta_{q}\right)\right)$$

and the range resolution is $\rho_{r}=c/\left(2\gamma T\right)$. For the targets with large rotors, the rotating radius $r_{q}$ might be much larger than the range resolution. Equation (8) indicates that the signal of the micromotion scatterer migrates through the range cells in the spectrogram and has the shape of a sinusiod. In contrast, the position of the main body signal is slow time invariant, which has the shape of a straight line. The sinusoid in the spectrogram will seriously affect the cross-range compression, i.e., the integration in the slow time domain, and lead to a smeared ISAR image. To remove the sinusoidal m-D interference, the method in Q. Zhang et al. [6] extracts the straight lines in the spectrogram using the Hough transform. Similarly, the methods in H.C. Liu et al. and L. Sun et al. [5,7] only recover the straight lines where the sinusoids are eliminated. However, when the target has small rotors, $r_{q}$ might be smaller than half of the range resolution. In this condition, the signal of the micromotion scatterer is located in a constant range cell and also has the shape of a straight line. The spectrogram can be approximately expressed as

(11) $$\begin{array}{l} s_{r}\left(\hat{f},\;t_{m}\right)\approx \sum_{p=1}^{P}A_{p}\sin c\left(T\left(\hat{f}+\frac{2\gamma }{c}\left(y_{p}-y_{u}\right)\right)\right)\exp\left(-j\frac{4\pi }{\lambda }\left(x_{p}\frac{v_{x}t_{m}}{R_{u}}+y_{p}-y_{u}\right)\right)\\ \;\;+\sum_{q=1}^{Q}A_{q}\sin c\left(T\left(\hat{f}+\frac{2\gamma }{c}\left(y_{o^{\prime }}-y_{u}\right)\right)\right)\exp\left(-j\frac{4\pi }{\lambda }\left(x_{o^{\prime }}\frac{v_{x}t_{m}}{R_{u}}+y_{o^{\prime }}-y_{u}+r_{q}\sin\left(\omega_{q}t_{m}+\theta_{q}\right)\right)\right). \end{array}$$

Because the main body signal and the signal of the micromotion scatterer have the same straight line shapes in the spectrogram, the methods in H.C. Liu et al., Q. Zhang et al., and L. Sun et al. [5,6,7] are invalid to remove the m-D effect. According to Equation (11), the Doppler frequencies of the main body scatterer and the micromotion scatterer are

(12) $$f_{p}=-\frac{2x_{p}v_{x}}{\lambda R_{u}}$$

(13) $$f_{q}=-\frac{2}{\lambda }\left(\frac{x_{o^{\prime }}v_{x}}{R_{u}}+r_{q}\omega_{q}\cos\left(\omega_{q}t_{m}+\theta_{q}\right)\right).$$

It is obvious from Equations (12) and (13) that the main body scatterer has a constant Doppler frequency while the Doppler frequency of the micromotion scatterer is slow time variant. Therefore, it is possible to separate the main body signal from the micromotion signal in the time-frequency domain.

## 3. Joint Sparsity of Main Body Signal in Time-Frequency Domain

Typical time-frequency transforms include the Wigner–Ville distribution (WVD) [27] and the STFT. Although WVD has better time-frequency resolution than STFT, it suffers from cross-terms for multiple signal components. Thus, we chose the STFT to achieve the time-frequency representations of the echoes in the contaminated range cell. In order to obtain the STFT entries, the echoes in the contaminated range cell were first segmented into narrow time intervals so that the signal in each segment could be considered stationary. Then, the Fourier transform was carried out for the windowed signal in each segment to obtain the local spectral representations. In this paper, we chose the rectangular window function and the segmentation of the echoes could be realized by a sliding window with proper time step.

**Figure 2:**
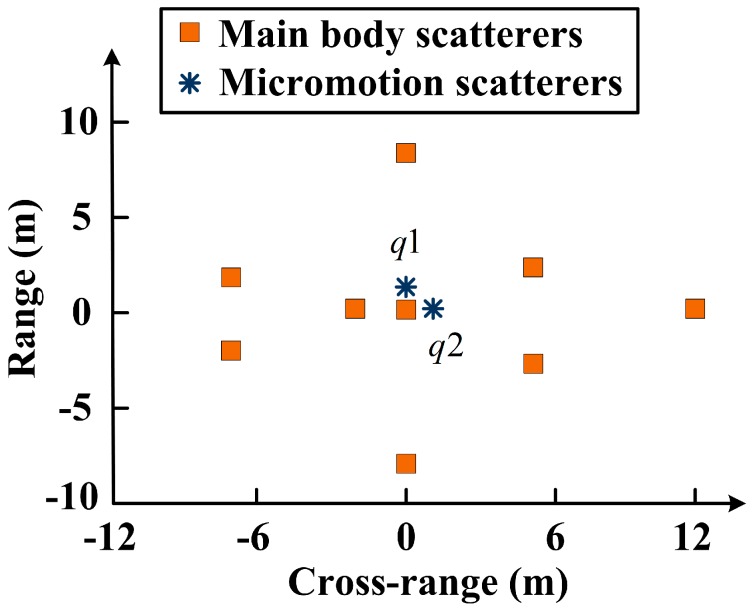
Target model.

To demonstrate the joint sparsity of the main body signal in the time-frequency domain, we conducted the following simulation. The target model was composed of nine main body scatterers and two micromotion scatterers, as described in Figure 2. The micromotion scatterers q1 and q2 rotated around the origin counterclockwise with radii of 0.1 m and 0.2 m, and with rotating frequencies of 10 Hz and 3 Hz, respectively. The initial rotating phases were π/2 rad and 0 rad, respectively. The target moved along the cross-range direction with a velocity of 300 m/s. The radar carrier frequency was 10 GHz, the bandwidth was 600 MHz, the pulse repetition interval (PRI) was 1 ms, and the CPI was 1 s.

**Figure 3:**
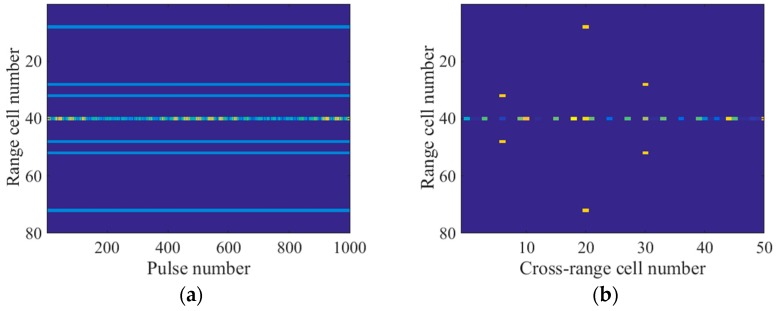
(**a**) Spectrogram; (**b**) Imaging result by the FFT-based method.

According to the radar system parameters, the range resolution was calculated to be 0.25 m, which was larger than twice the rotating radii of both q1 and q2. Thus, the micromotion signal did not migrate through the range cells in the spectrogram. As shown in Figure 3a, the micromotion signal was located in the 40th range cell and had the same straight line shape as the main body signal. In this situation, the methods in H.C. Liu et al., Q. Zhang et al., and L. Sun et al. [5,6,7] were invalid to eliminate the micromotion components. As a result from the m-D effect, the application of a fast Fourier transform (FFT) to the spectrogram along the slow time dimension would lead to a blurred main body image, as depicted in Figure 3b. We selected 50 cross-range cells where the target was located in the imaging result to display.

**Figure 4:**
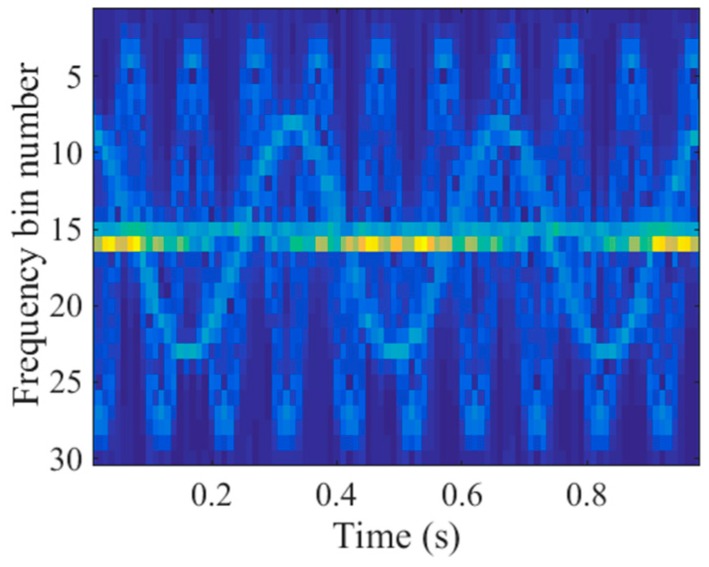
Short-time Fourier transform (STFT) entries of the echoes in the 40th range cell.

To obtain the time-frequency representations, we applied the STFT to the echoes in the 40th range cell of the spectrogram. The time step was 10 PRI and the window width was 30 PRI. Thus, there were 30 frequency bins in the time-frequency domain. As shown in Figure 4, the main body signal was located at the 15th and 16th frequency bins. Consequently, the support of the main body signal in the time-frequency domain was slow time invariant which indicated the joint sparsity of the main body signal. In contrast, the micromotion scatterers’ Doppler frequencies were slow time variant and the micromotion signal had the shape of sinusoids, as illustrated in Figure 4. Because of the distinct patterns, the micromotion signal could be removed by promoting the joint sparsity in the time-frequency domain, which is detailed in the next section.

## 4. M-D Effect Removal by Promoting Joint Sparsity

### 4.1. Problem Formulation for Joint Sparse Recovery

The echoes in the range cell contaminated by the m-D effect are denoted by s, and we assume that the number of the received pulses is V. The window width and the time step of the STFT are denoted by N and G, respectively. Thus, the number of the segments in the STFT is M=⌈(V−N)/G⌉+1, where ⌈⋅⌉ denotes rounding to the nearest integer towards infinity.

The discrete Fourier matrix for each segment is

(14) $$Q={\left[\begin{array}{cccc} 1 & 1 & \cdots & 1\\ 1 & W^{-1} & \cdots & W^{-\left(N-1\right)}\\ \vdots & \vdots & \ddots & \vdots \\ 1 & W^{-\left(N-1\right)} & \cdots & W^{-\left(N-1\right)\left(N-1\right)} \end{array}\right]}_{N\times N}$$

where W=exp(j2π/N). Denoting the STFT entries in Figure 4 by X, the corresponding STFT entry vector is x, which is generated by stacking the columns of X into a single vector. Then, the STFT of s can be written in a compact matrix form

(15) x=Hs

where $H\;\in \;{\mathbb{C}}^{K\times V}$ denotes the STFT matrix

(16) $$H={\left[\begin{array}{lll} Q & & 0_{N\times \left(V-N\right)}\\ 0_{N\times G} & Q & 0_{N\times \left(V-G-N\right)}\\ 0_{N\times 2G} & Q & 0_{N\times \left(V-2G-N\right)}\\ \vdots & \vdots & \vdots \\ 0_{N\times \left(V-N\right)} & & Q \end{array}\right]}_{K\times V}$$

and K=MN is the number of the STFT entries in x. $0_{N\times G}$ denotes a matrix of size N×G, whose elements are equal to 0.

The echoes in the contaminated range cell can be modeled as follows:

(17) s=Dx+ϕ

where $\phi \in {\mathbb{C}}^{V\times 1}$ denotes the noise, and $D={\left(H^{H}H\right)}^{-1}H^{H}$ is the Moore–Penrose inverse of the STFT matrix H. However, the data samples of the echoes might be randomly missing in real world scenarios where strong electromagnetic interference or sensor failure prevents effective observation. The data samples under strong electromagnetic interference must be discarded and the remaining L samples constitute the measurement vector $y\in {\mathbb{C}}^{L\times 1}$, which can be expressed as

(18) y=Ax+n

where $n\in {\mathbb{C}}^{L\times 1}$ is the noise. $A\in {\mathbb{C}}^{L\times K}$ is formed by omitting the rows of D which correspond to the missing samples. Because the remaining samples are fewer than the STFT entries to recover, i.e., L<K, Equation (18) is underdetermined.

To facilitate the notation, we rearrange the STFT entries in the ith frequency bin into a vector

(19) $$b_{i}=\left[x_{i},x_{N+i},\;\ldots ,\;x_{MN+i}\right],\;1\leq i\leq N.$$

According to the analysis in Section 3, the micromotion components in the time-frequency domain can be eliminated by promoting the joint sparsity of x. The joint sparse recovery problem is usually formulated by the following $l_{2}/l_{0}$ minimization problem

(20) $$\hat{x}=\arg\;\underset{x}{\min}\;{\left\|x\right\|}_{2,0}\;s.t.\;{\left\|y-Ax\right\|}_{2}<\varepsilon$$

where ε bounds the $l_{2}$-norm of the noise in the measurements. ${\left\|x\right\|}_{2,0}$ stands for the $l_{2}/l_{0}$-norm of x defined as [16]

(21) $${\left\|x\right\|}_{2,0}={\left\|\beta \right\|}_{0}$$

where $\beta ={\left[{\left\|b_{1}\right\|}_{2},{\left\|b_{2}\right\|}_{2},\ldots ,{\left\|b_{N}\right\|}_{2}\right]}^{T}$. It is obvious that ${\left\|x\right\|}_{2,0}$ is equivalent to the number of frequency bins which have nonzero STFT entries. Therefore, by solving the problem in Equation (20), we can find the solution $\hat{x}$ which occupies the fewest frequency bins and the joint sparsity in the time-frequency domain can be promoted.

However, the problem in Equation (20) is essentially a combinatorial optimization problem [17], which is difficult to solve. Inspired by the smoothed $l_{0}$ (SL0) algorithm, we propose a novel objective function to replace ${\left\|x\right\|}_{2,0}$, so that the new problem is tractable. In the following subsections, the original SL0 algorithm is briefly reviewed at first. Then, we elaborate the proposed ISAR imaging method.

### 4.2. Brief Review of SL0 Algorithm

The basic idea of the SL0 algorithm is to approximate the $l_{0}$-norm of x with the following function [23]

(22) $$F_{\sigma }\left(x\right)=\sum_{i=1}^{MN}f_{\sigma }\left(x_{i}\right)$$

where

(23) $$f_{\sigma }\left(x\right)=\exp\left(-\frac{x^{2}}{2\sigma^{2}}\right)$$

and

(24) $$\underset{\sigma \to 0}{\lim}\;\;\;f_{\sigma }\left(x\right)=\left\{ \begin{array}{l} 1,\;\mathrm{if}\;\;\;\left(x=0\right)\\ 0,\;\mathrm{if}\;\;\;\;\left(x\neq 0\right) \end{array}\right.$$

It can be seen from Equations (22) and (24) that ${\left\|x\right\|}_{0}\approx MN-F_{\sigma }\left(x\right)$ for small values of σ. Furthermore, this approximation becomes equality as σ→0. The SL0 algorithm obtains the sparse solution of Equation (18) by solving the following problem

(25) $$\tilde{x}=\arg\;\underset{x}{\max}\;F_{\sigma }\left(x\right)\;s.t.\;{\left\|y-Ax\right\|}_{2}\leq \varepsilon .$$

To avoid trapping into local maxima, a decreasing sequence of σ is utilized, i.e., $\left[\sigma_{1},\ldots ,\sigma_{\eta }\right]$, where $\sigma_{i}>\sigma_{j}$ for i>j. η is the iteration number and the current maximizer of $F_{\sigma }\left(x\right)$ is used as the starting point of the next iteration. To maximize $F_{\sigma }\left(x\right)$ for a fixed σ, there are two steps in each iteration: the unconstrained maximization step $x^{\prime }\leftarrow x+\mu \nabla F_{\sigma }\left(x\right)$ and the projection to the feasible set

(26) $$\begin{array}{ll} x & =\arg\;\underset{x}{\min}\;{\left\|x-x^{\prime }\right\|}_{2}\;s.t.\;y=Ax\\ & =x^{\prime }-A^{†}\left(Ax^{\prime }-y\right) \end{array}$$

where $A^{†}=A^{H}{\left(AA^{H}\right)}^{-1}$, and $\nabla F_{\sigma }\left(x\right)$ is the gradient of $F_{\sigma }\left(x\right)$.

### 4.3. Proposed Method

Although it has been shown in H. Mohimani et al. [23] that the SL0 algorithm is effective to obtain the sparse solution of Equation (18), the joint sparsity of x cannot be promoted. It can be seen from Equation (22) that the SL0 algorithm only promotes the sparsity of the individual entries in x, and $\left(MN-F_{\sigma }\left(x\right)\right)$ is merely able to approximate ${\left\|x\right\|}_{0}$ rather than ${\left\|x\right\|}_{2,0}$, which measures the joint sparsity of x. As a result, the SL0 algorithm is inappropriate to remove the m-D effect in ISAR imaging for micromotion targets. In this paper, we propose a smoothed $l_{2}/l_{0}$ (SL2L0) algorithm, where ${\left\|x\right\|}_{2,0}$ in Equation (20) is approximated by a new objective function

(27) $$\begin{array}{ll} U_{\sigma }\left(x\right) & =N-\sum_{i=1}^{N}f_{\sigma }\left({\left\|b_{i}\right\|}_{2}^{2}\right)\\ & =N-\sum_{i=1}^{N}\exp\left(-\frac{\sum_{j=1}^{M}{\left|x_{\left(j-1\right)N+i}\right|}^{2}}{2\sigma^{2}}\right). \end{array}$$

Since ${\left\|x\right\|}_{2,0}$ is equivalent to the number of frequency bins which have nonzero STFT entries, it is obvious from Equations (19), (24) and (27) that $U_{\sigma }\left(x\right)={\left\|x\right\|}_{2,0}$ when σ→0. Therefore, the joint sparsity can be promoted by minimizing the new objective function $U_{\sigma }\left(x\right)$ with small values of σ. The joint sparse recovery problem can be formulated as

(28) $$\bar{x}=\arg\;\underset{x}{\min}\;U_{\sigma }\left(x\right)\;s.t.\;{\left\|y-Ax\right\|}_{2}\leq \varepsilon .$$

Similar to the SL0 algorithm, a decreasing sequence of σ is used to avoid trapping into local minimum and a steepest descend approach is employed to minimize $\;U_{\sigma }\left(x\right)$ in each iteration, i.e.,

(29) $$x^{\prime }=x-\left(2\mu \sigma^{2}\right)\nabla \;U_{\sigma }\left(x\right)$$

where μ is the parameter controlling the step-size $\left(2\mu \sigma^{2}\right)$. The gradient $\nabla \;U_{\sigma }\left(x\right)$ in Equation (29) is calculated to be

(30) $$\nabla \;U_{\sigma }\left(x\right)=1_{N}⊗\alpha ⊙x$$

where $1_{N}$ is a vector of length N with its entries equal to 1, ⊗ is the Kronecker product, ⊙ is the Hadamard product, and $\alpha \in {\mathbb{C}}^{N\times 1}$ is

(31) $$\alpha ={\left[\frac{1}{2\sigma^{2}}\exp\left(-\frac{\sum_{j=1}^{M}{\left|x_{\left(j-1\right)N+1}\right|}^{2}}{2\sigma^{2}}\right)\;\cdots \;\frac{1}{2\sigma^{2}}\exp\left(-\frac{\sum_{j=1}^{M}{\left|x_{\left(j-1\right)N+N}\right|}^{2}}{2\sigma^{2}}\right)\right]}^{T}.$$

Since the micromotion components are eliminated by promoting the joint sparsity of x, we modify the projection step in Equation (26) as follows:

(32) $$x=\arg\;\underset{x}{\min}\;{\left\|x-x^{\prime }\right\|}_{1}\;s.t.\;{\left\|y-Ax\right\|}_{2}\leq \lambda$$

where λ bounds the $l_{2}$-norm of the micromotion components in the echoes. Equation (32) can be rewritten into an unconstrained optimization problem

(33) $$z=\arg\;\underset{z}{\min}\;\frac{1}{2}{\left\|y^{\prime }-Az\right\|}_{2}+\tau {\left\|z\right\|}_{1}$$

where $z=x-x^{\prime }\;$ and $y^{\prime }=y-Ax^{\prime }$. τ is the regularization parameter related to λ. $g\left(z\right)={\left\|z\right\|}_{1}$ is called the regularizer which indicates that the optimization problem in Equation (33) is convex. We use the fast iterative shrinkage-thresholding algorithm (FISTA) [28,29] to solve the problem in Equation (33) efficiently. The key concept in FISTA is known as Moreau’s proximal operator, or proximal operator for short, which is expressed as

(34) $${\mathrm{prox}}_{\theta g}\left(z_{k}\right)=\frac{z_{k}}{\left|z_{k}\right|}\max\left(\left|z_{k}\right|-\theta \tau ,0\right),\;1\leq k\leq K\;,$$

and θ in Equation (34) is a positive constant with ${\theta \geq 1/\left\|A\right\|}_{F}^{2}$.

In summary, the successive steps of the proposed SL2L0 algorithm are given in Algorithm 1.

**Algorithm 1** Proposed smoothed $l_{2}/l_{0}$ (SL2L0) algorithm**Input:**The echo sampling vector y, the overcompleted dictionary A, positive constants {μ,θ,ε}, the maximum number of outer loop iterations η, the maximum number of inner loop iterations ϕ, a suitable decreasing sequence $\left[\sigma_{1}\cdots \sigma_{\eta }\right]$, the tolerance values ez and ex; initialization $x^{\left(1\right)}=A^{†}y$ and $t^{\left(1\right)}=1$.**Iteration:**
For i=1,…,η   $\sigma =\sigma_{i}$;   $x^{\prime }=x^{\left(i\right)}-\left(2\mu \sigma^{2}\right)\nabla \;U_{\sigma }\left(x^{\left(i\right)}\right)$;   $y^{\prime }=y-Ax^{\prime }$;   $z^{\left(0\right)}=x^{\left(i\right)}-x^{\prime }$;   $q^{\left(1\right)}=z^{\left(0\right)}$;   For j=1,⋯,ϕ      $z^{\left(j\right)}={\mathrm{prox}}_{\theta g}\left(q^{\left(j\right)}-\theta A^{H}\left(Aq^{\left(j\right)}-y^{\prime }\right)\right)$;      $t^{\left(j+1\right)}=\frac{1+\sqrt{1+4t^{\left(j\right)}}}{2}$;      $q^{\left(j+1\right)}=z^{\left(j\right)}+\frac{t^{\left(j\right)}-1}{t^{\left(j+1\right)}}\left(z^{\left(j\right)}-z^{\left(j-1\right)}\right)$;      if ${{\left\|z^{\left(j\right)}-z^{\left(j-1\right)}\right\|}_{2}/\left\|z^{\left(j-1\right)}\right\|}_{2}<ez$, then break;   End   $x^{\left(i+1\right)}=x^{\prime }+z^{\left(j\right)}$;   if $\;{\left\|y-Ax^{\left(i+1\right)}\right\|}_{2}<\varepsilon$ or ${{\left\|x^{\left(i+1\right)}-x^{\left(i\right)}\right\|}_{2}/\left\|x^{\left(i\right)}\right\|}_{2}<ex$, then break; End**Output:**
$\bar{x}=x^{\left(i+1\right)}$

By promoting the joint sparsity in the time-frequency domain, the output $\bar{x}$ of the SL2L0 algorithm only contains the main body components. Based on the recovered STFT entries, we can obtain the main body echoes free of m-D effect as $\bar{y}=A\bar{x}$. Assuming that the residual noise in $\bar{y}$ is $e\in {\mathbb{C}}^{L\times 1}$, $\bar{y}$ can be rewritten as

(35) $$\bar{y}=\Omega p+e$$

where $p\in {\mathbb{C}}^{V\times 1}$ is the cross-range compression result of $\bar{y}$, and $\Omega \in {\mathbb{C}}^{L\times V}$ is a partial Fourier matrix with its (l,v)th element given by $\exp\left(-j2\pi f_{v}t_{l}\right)$. $f_{v}$ is the vth Doppler frequency bin, and $t_{l}$ is the lth sampling point of the slow time. Subsequently, we used the sparse Bayesian learning (SBL) [25] algorithm to achieve the cross-range compression of $\bar{y}$ by recovering p in Equation (35).

Although the discussions in this section are focused on the echoes in a single range cell, the proposed method is also suitable for other contaminated range cells. After all of the contaminated range cells are processed by the proposed method, a clear main body image can be achieved.

## 5. Experimental Results and Performance Comparisons

In this section, we discuss the experiments conducted using the echoes generated by the point-scattering model and EM computation, respectively. To validate the effectiveness, the imaging results of the proposed method and other existing methods are compared. The parameters of the proposed method used in the experiments are set as follows: μ=1, $\theta ={1/\left\|A\right\|}_{F}^{2}$, η=40, ϕ=20, $\sigma_{i}=0.8^{i-1}$ for i=1,…,η, $ez=10^{-4}$, and $ex=10^{-4}$. Since ε is related to the noise level, this parameter is tuned manually in each experiment.

### 5.1. Experiments Using the Point-Scattering Model

The target model is depicted in Figure 2, and the radar system parameters are the same as in Section 3. We obtained the limited measurements by choosing 500 pulses randomly in the slow time domain, and the signal-to-noise ratio (SNR) was 20 dB. The number of cross-range cells to be recovered was 1000. The window width of the STFT was 30 PRI and the time step was 10 PRI. According to the above parameters, the size of A was calculated to be 500×2940. Similar to Figure 3b, we selected 50 cross-range cells to display in the imaging results.

**Figure 5:**
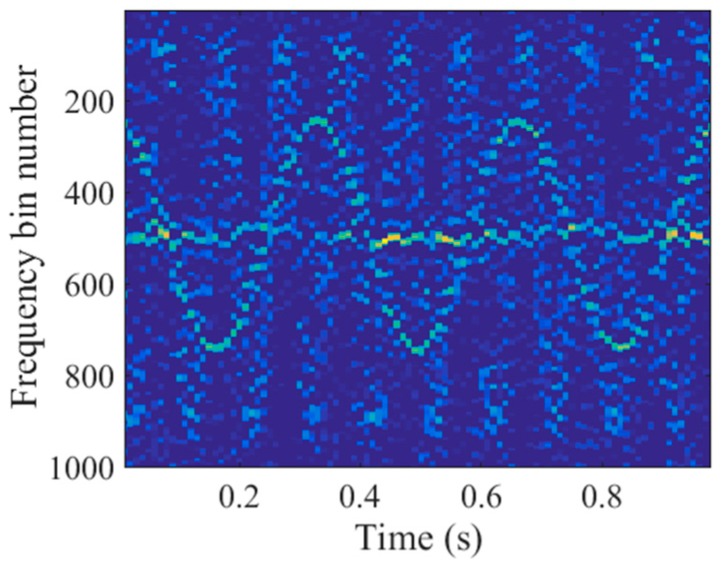
Time-frequency representations obtained by the short-time compressed sensing (STCS).

**Figure 6:**
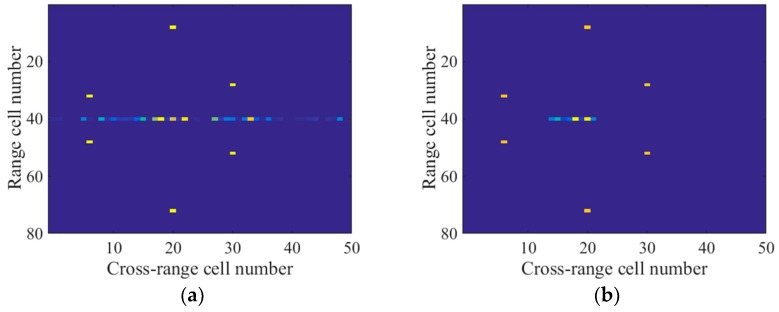
(**a**) Imaging result by the method in [9]; (**b**) Imaging result by the L-statistics-based method.

**Figure 7:**
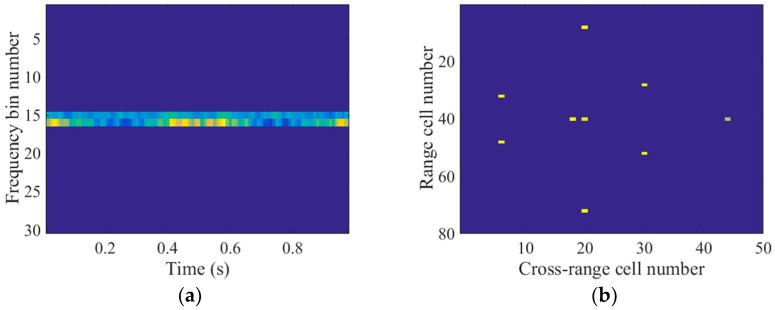
(**a**) STFT entries recovered by the SL2L0 algorithm; (**b**) Imaging result by the proposed method.

To eliminate the micromotion components of the echoes in the contaminated range cell, both the method in R. Zhang et al. [9] and the L-statistics-based method utilize the time-frequency representations obtained by the STCS [11]. However, part of the main body signal in the time-frequency domain is missing due to the discarded pulses, as illustrated in Figure 5. Additionally, there is a strong m-D signal which has the shape of sinusoids. As a result, the histogram analysis in R. Zhang et al. [9] could not be used to remove the m-D signal, which might have been mistaken for the main body signal. Figure 6a shows the imaging result produced by the method in R. Zhang et al. [9]. Many spurious points exist in the 40th range cell due to the m-D interference and the main body scatterers are defocused. Because the measurements within the sliding window are much fewer than the frequency bins in STCS, the frequency resolution in the time-frequency domain is lower than the reciprocal of the CPI. Therefore, the L-statistics-based method cannot obtain the accurate support of the main body scatterers in the cross-range domain. As depicted in Figure 6b, some spurious points exist near the main body scatterers. In addition, one of the main body scatterers in the 40th range cell cannot be recovered because part of the main body signal is missing in Figure 5. In contrast to STCS, the proposed SL2L0 algorithm can remove the m-D signal by promoting the joint sparsity in the time-frequency domain, where only the main body signal with constant Doppler frequencies are preserved, as shown in Figure 7a. Based on the recovered STFT entries, the proposed method achieves a clear main body image in Figure 7b.

In addition to the visual results, we introduce the normalized mean square error (NMSE) and the image contrast (IC) as two metrics to compare the performance of different methods. A lower NMSE means the image has less recovery error, and a higher IC indicates the image is better focused. The NMSE is computed by [30]

(36) $$\mathrm{NMSE}=\frac{1}{U}{\sum_{k=1}^{U}\left\|\frac{I}{{\left\|I\right\|}_{F}}-\frac{{\hat{I}}_{k}}{{\left\|{\hat{I}}_{k}\right\|}_{F}}\right\|}_{F}$$

where U=100 independent trials are conducted, and ${\hat{I}}_{k}\in {\mathbb{C}}^{R\times V}$ denotes the recovered result of the true main body image I in the kth trial. The IC is computed by X. Z. Gao et al. [24]

(37) $$\mathrm{IC}=\frac{\sqrt{Ave\left({\left({\hat{I}}_{k}^{2}-Ave\left({\hat{I}}_{k}^{2}\right)\right)}^{2}\right)}}{Ave\left({\hat{I}}_{k}^{2}\right)}$$

where ${\hat{I}}_{k}^{2}$ represents the intensity matrix of the recovered main body image with its (r,v)th element ${\hat{I}}_{k}^{2}\left(r,v\right)$ equal to ${\left|{\hat{I}}_{k}\left(r,v\right)\right|}^{2}$. Ave(⋅) denotes the mean operator, which is defined as

(38) $$Ave\left(I\right)=\frac{1}{RV}\sum_{r=1}^{R}\sum_{v=1}^{V}I\left(r,v\right)$$

**Figure 8:**
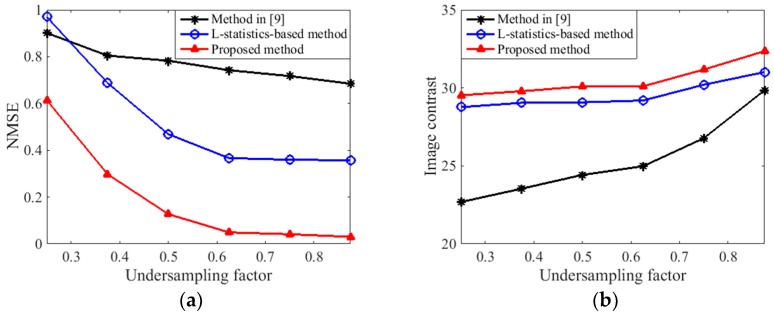
Performance of different methods: (**a**) Normalized mean square error (NMSE) against undersampling factor; (**b**) Image contrast (IC) against undersampling factor.

Figure 8 gives the NMSEs and ICs obtained by different methods against the undersampling factor [31], which is calculated as ψ=L/V, i.e., the number of measurements in slow time dimension divided by the number of cross-range cells to recover. For each ψ, the IC is averaged over 100 independent trials. It can be observed from Figure 8 that the proposed method achieves the lowest NMSEs and the highest ICs among all of the methods, which indicate the least recovery error and the best focused quality, respectively.

### 5.2. Experiments Using EM Computation

**Figure 9:**
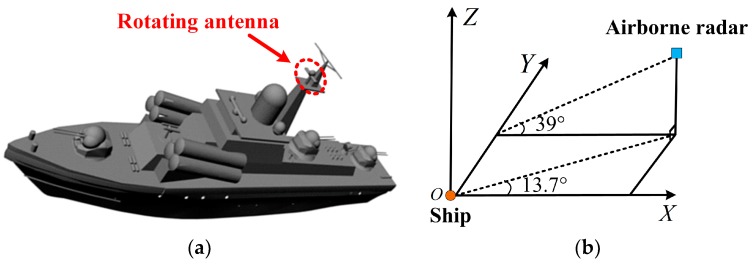
(**a**) Ship model; (**b**) Imaging geometry.

To further testify the performance of the proposed method, we used the echoes of a ship target generated by the graphic electromagnetic computing (GRECO) technique [32], which combines physical optical (PO) theory and physical theory of diffraction (PTD). The target model is described in Figure 9a, where the dashed circle indicates the antenna rotating with a frequency of 1 Hz. Figure 9b depicts the imaging geometry. The ship was 6.9 km from the airborne radar and located at the origin. The radar operated at 5 GHz, the bandwidth was 1 GHz, and the radar moved along the opposite direction of the X-axis with a constant velocity of 300 m/s. 256 pulses were collected in a CPI of 2.3 s and the SNR was 20 dB. We obtained the limited measurements by choosing 128 pulses randomly. Within each pulse, there were 128 complex range samples. The window width and the time step of the STFT were 20 PRI and 2 PRI, respectively. According to the aforementioned parameters, the size of A was calculated to be 128×2380.

**Figure 10:**
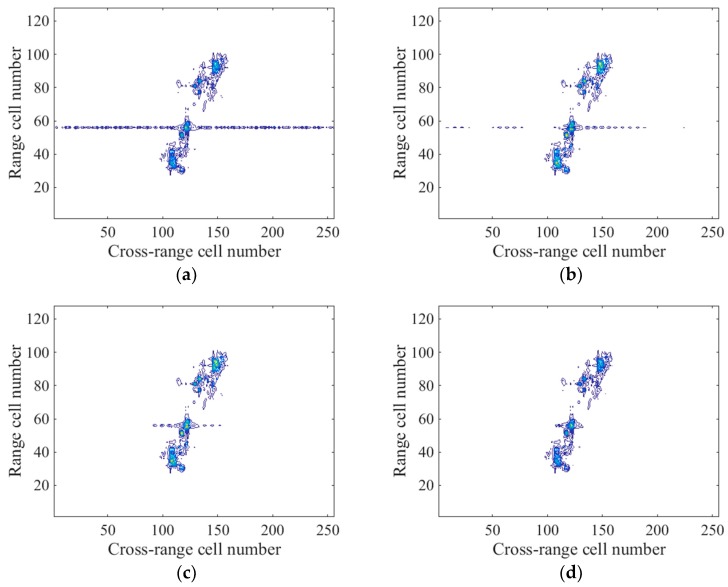
(**a**) Imaging result by the FFT-based method; (**b**) Imaging result by the method in R. Zhang et al. [9]; (**c**) Imaging result by the L-statistics-based method; (**d**) Imaging result by the proposed method.

The imaging results by different methods are shown in Figure 10. It can be seen from Figure 10a that the m-D effect led to a blurred main body image by the FFT-based method. Though the method in R. Zhang et al. [9] and the L-statistics-based method suppressed the m-D effect to some degree, there were residuals and some spurious points exist in Figure 10b,c. In contrast, the proposed method could remove the m-D effect through the SL2L0 algorithm and a clear image with the least spurious points is achieved in Figure 10d.

To quantitatively evaluate the performance of different methods, we used the image entropy (IE). Generally, a smaller IE indicates a better focused quality of the image. The IE is defined by W. Qiu et al. [33]

(39) $$\mathrm{IE}=-\sum_{r=1}^{R}\sum_{v=1}^{V}\bar{I}\left(r,v\right)\ln\left[\bar{I}\left(r,v\right)\right]$$

where $\bar{I}$ is the power normalized image computed by

(40) $$\bar{I}=\frac{I^{2}}{\sum_{r=1}^{R}\sum_{v=1}^{V}{\left|I\left(r,v\right)\right|}^{2}}$$

The IEs of the imaging results are given in Table 1. The proposed method achieved the minimum IE which verifies the superiority of the proposed method to other methods.

The computational time of different methods was recorded based on the MATLAB code and summarized in Table 2, where the results are given by the averages of 50 independent trials conducted on an Intel Core i7 machine at 3.50 GHz. The computational time of the FFT-based method was not recorded since it could not suppress the m-D effect, as shown in Figure 10a. It can be seen from Table 2 that the proposed method is more computationally efficient than the method in R. Zhang et al. [9] and the L-statistics-based method.

## 6. Conclusions

In ISAR imaging, the m-D effect generated by the micromotion scatterers with small rotating radii leads to a blurred main body image. It is claimed that the Doppler frequency of the micromotion scatterer is slow time variant while the main body scatterer has a constant Doppler frequency, indicating the joint sparsity property in the time-frequency domain. To remove the m-D effect, this paper proposes a novel algorithm named SL2L0, where a new objective function is used to measure the joint sparsity and is minimized in a way similar to the SL0 algorithm. We have tailored the projection step in the SL0 algorithm to make it suitable for m-D effect removal. Experimental results showed that the proposed SL2L0 algorithm can effectively remove the m-D effect by promoting the joint sparsity in the time-frequency domain and a clear main body image can be achieved after cross-range compression. Diverse metrics, including the NMSE, IC, and IE, were employed to validate the superiority of the proposed method to other existing methods.

## Figures and Tables

**Table 1:** Performance Evaluation by Image Entropy.

Method	Image Entropy
The FFT-based method	5.977
The method in [9]	4.865
The L-statistics-based method	4.803
The proposed method	4.204

**Table 2:** Computational Time.

Method	Computational Time (s)
The method in [9]	5.529
The L-statistics-based method	3.406
The proposed method	1.811
